# Computed tomography enterography increases the ability of endoscopy to differentiate Crohn's disease from intestinal Behçet's disease

**DOI:** 10.3389/fmed.2022.900458

**Published:** 2022-08-18

**Authors:** Hong Yang, Huimin Zhang, Wei Liu, Wei Han, Tao Guo, Yamin Lai, Bei Tan, Congling Wang, Minhu Chen, Xiang Gao, Zhihua Ran, Zhanju Liu, Kaichun Wu, Qian Cao, Jiaming Qian

**Affiliations:** ^1^Department of Gastroenterology, Peking Union Medical College Hospital, Chinese Academy Medical Sciences and Peking Union Medical College, Beijing, China; ^2^Department of Radiology, Peking Union Medical College Hospital, Chinese Academy Medical Sciences and Peking Union Medical College, Beijing, China; ^3^Department of Epidemiology and Biostatistics, Institute of Basic Medical Sciences, Peking Union Medical College, Chinese Academy of Medical Sciences, School of Basic Medicine, Beijing, China; ^4^Department of Gastroenterology, The First Affiliated Hospital of Sun Yat-sen University, Guangzhou, China; ^5^Department of Gastroenterology, The Sixth Affiliated Hospital of Sun Yat-sen University, Guangzhou, China; ^6^Division of Gastroenterology and Hepatology, Key Laboratory of Gastroenterology and Hepatology, Ministry of Health, Shanghai Inflammatory Bowel Disease Research Center, Renji Hospital, School of Medicine, Shanghai Jiao Tong University, Shanghai, China; ^7^Department of Gastroenterology, The Shanghai Tenth People's Hospital, Tongji University, Shanghai, China; ^8^State Key Laboratory of Cancer Biology, National Clinical Research Center for Digestive Diseases and Xijing Hospital of Digestive Diseases, Fourth Military Medical University, Xi'an, China; ^9^Department of Gastroenterology, Sir Run Run Shaw Hospital, College of Medicine Zhejiang University, Hangzhou, China

**Keywords:** Crohn's disease, Behçet's disease, diagnosis, scoring model, endoscopy

## Abstract

**Background:**

Distinguishing Crohn's disease (CD) and intestinal Behçet's disease (BD) is difficult in clinical practice.

**Aim:**

To evaluate the ability of CT enterography (CTE) to enhance the diagnostic value of endoscopy in differentiating CD from intestinal BD and to establish differential diagnosis models.

**Methods:**

A total of 113 patients with CD and 70 patients with intestinal BD from seven tertiary inflammatory bowel disease centers were enrolled. The univariate and multivariate analyses were used by SAS software version 9.2. Three differential scoring models based on the multivariate analysis of endoscopic features alone (model 1), endoscopic features combined with clinical symptoms (model 2), and endoscopic features combined with clinical symptoms and CTE (model 3) were established.

**Results:**

The results showed that model 2 increased the efficacy of model 1 in differential diagnosis and model 3 had the highest accuracy of 84.15% at a cutoff value of two points. The scoring of model 3 was as follows: genital ulcer (−3 points), skin lesions (−3 points), oval ulcer (-2 points), longitudinal ulcer (1 point), number of ulcers > 5 (3 points), inflammatory polyps (2 points), mucosal severe enhancement (2 points), and fibrofatty proliferation (1 point).

**Conclusion:**

Clinical symptoms and CTE increased the ability of endoscopy to differentiate CD from intestinal BD.

## Highlights

What is known:

- Crohn's disease (CD) and intestinal Behçet's disease (BD) have many similarities and differential diagnosis is difficult.
- Crohn's disease and intestinal BD have different prognoses.

What is new here:

- Endoscopic and imaging indicators significantly improved the ability to differentiate between CD and intestinal BD.

This model in our study is useful to differentiate CD from intestinal BD.

## Introduction

Crohn's disease (CD) is a chronic and recurrent inflammatory disorder with the appearance of intestinal and colon ulcerations, which is difficult to distinguish from intestinal Behçet's disease (BD). BD is a chronic recurrent systemic inflammatory disease, typically presenting as oral and genital ulcerations and ocular lesions. Intestinal BD, the incidence of which accounts for 5–25% of BD, is diagnosed when typical intestinal ulcerations exist (1, 2). CD and intestinal BD have similar clinical characteristics, so it remains a tough problem in the differential diagnosis. The choice of immunosuppressants for intestinal BD complicated with the involvement of different organs may be different. For example, colchicine can be used for intestinal BD complicated with skin and mucous membrane involvement, and cyclophosphamide can be used for intestinal BD complicated with nervous system involvement (3). However, all the treatments mentioned above have limited effects in patients with CD. In addition, it has been reported that patients with intestinal BD are more prone to intestinal perforation and intestinal bleeding and the mortality rate of patients with intestinal BD is higher compared with that of patients with CD (4, 5). So, early and accurate diagnosis is very important.

To date, there are few studies on the differential diagnosis of CD and intestinal BD. Lee et al. compared and analyzed the endoscopic characteristics of the two diseases and established a differential diagnosis model, including ulcer shape and distribution with an accuracy of 92% (6). However, the accuracy rate of this model was only 73% when applied to Chinese patients (4). Seeking a method suitable for discriminating CD from intestinal BD in China is a prerequisite.

Computed tomography enterography (CTE) is a newly developed technique that can disclose the extent of the lesion involved, visualize the thickening of the intestinal wall, and reveal the characteristics of the mucosal and serous surfaces and mesentery around the lesion. CTE can also detect small intestinal lesions that cannot be observed by colonoscopy. There are few studies on the value of CTE to differentiate CD from intestinal BD. This study aimed to compare and evaluate the accuracy of endoscopic features alone and the enhanced effect of combining clinical symptoms, and endoscopic and CTE features in differentiating the two diseases. Furthermore, this study was also designed to establish the differential scoring models with the expectation that the models can be applied to clinical practice for precise diagnosis and treatment.

## Materials and methods

### Patients

A total of 70 patients with intestinal BD and 113 patients with CD with clinical, endoscopic, and CTE data who were diagnosed and treated in the Peking Union Medical College Hospital, the First Affiliated Hospital of Sun Yat-Sen University, the Sixth Affiliated Hospital of Sun Yat-Sen University, Renji Hospital Affiliated to Shanghai Jiaotong University, Shanghai Tenth People's Hospital, Xijing Hospital, and the Sir Run Run Shaw Hospital from 1 January 2004 to 30 June 2018 were enrolled in the study. This study was approved by the Institutional Review Board of Peking Union Medical College Hospital (No. S-K1101).

#### Inclusion and exclusion criteria

The inclusion criteria were as follows: (1) patients with clinically or pathologically confirmed CD or intestinal BD and (2) patients with complete clinical, endoscopic, and CTE data. Endoscopic and CTE data included the original images and reports.

The exclusion criteria included the following: (1) postsurgery or posttreatment patients and (2) patients with an uncertain diagnosis.

### Diagnostic criteria of Crohn's disease and intestinal Behçet's disease

The diagnosis of CD was reviewed and confirmed according to the European Crohn's and Colitis Organization (ECCO) and Chinese consensus based on clinical manifestations, endoscopic and CTE features, or pathological features (7, 8).

The diagnosis of intestinal BD was based on the pathological diagnosis or the diagnostic criteria developed by Cheon et al. (9). Confirmed diagnosis, probable diagnosis, and suspected diagnosis according to Cheon's study were all enrolled in this study according to extraintestinal manifestations and endoscopic intestinal ulcers.

Among the 70 patients with intestinal BD, three intestinal BD cases were pathologically confirmed, nine intestinal BD cases were clinically confirmed, 36 intestinal BD cases were clinically diagnosed as probable, and 22 intestinal BD cases were clinically suspected. There were no statistical significances in the CTE features among the confirmed, probable, and suspected groups (Table 1), further ensuring the consistency among the three subgroups as much as possible. For patients with a probable clinical diagnosis or suspected diagnosis, clinical follow-up was conducted for at least 1 year, which was consistent with the course of intestinal BD, and there was no evidence of other inflammatory diseases.

**Table 1:** Comparison of CTE features among subgroups of intestinal BD.

**Variables**	**Confirmed intestinal BD (*n* = 12)**	**Probable intestinal BD (*n* = 36)**	**Suspected intestinal BD (*n* = 22)**	*p-*values*
**Length of the longest lesion segment**				0.660
<5 cm	6 (50.0%)	14 (38.9%)	9 (40.9%)	
5–10 cm	4 (33.3%)	14 (38.9%)	8 (36.4%)	
10–30 cm	1 (8.3%)	4 (11.1%)	5 (22.7%)	
>30 cm	1 (8.3%)	4 (11.1%)	0 (0)	
Lesion thickness <1 cm	12 (100.0%)	35 (97.2%)	36 (100.0%)	1.000
**Enhancement degree**				0.256
Mild	5 (41.7%)	17 (47.2%)	10 (45.5%)	
Moderate	4 (33.3%)	18 (50.0%)	10 (45.5%)	
Severe	3 (25.0%)	1 (2.8%)	2 (9.1%)	
Asymmetric mural hyperenhancement	5 (41.7%)	15 (41.7%)	3 (13.6%)	0.061
Stratified mural hyperenhancement	11 (91.7%)	31 (86.1%)	21 (95.5%)	0.663
Polypoid lesions of mucosal surface	2 (16.7%)	11 (30.6%)	11 (50.0%)	0.139
Fibrofatty proliferation	4 (33.3%)	18 (50.0%)	9 (40.9%)	0.586
Engorged vasa recta	2 (16.7%)	8 (22.2%)	8 (36.4%)	0.463

* χ2 test or Fisher's exact test is used.

### Data collection

#### Demographic and clinical data

Demographic and clinical data included patient sex, age at the onset of gastrointestinal symptoms, clinical presentations, personal and past history, extraintestinal manifestations, and complications (shown in Tables 2, 3).

**Table 2:** Demographic characteristics of participants with CD or intestinal BD.

**Characteristics**	**Intestinal BD (*n* = 70)**	**CD** **(*n* = 113)**	*p*–values*
Gender, Men, *n* (%)	40 (57.1%)	84 (74.3%)	<0.0001
Onset age, mean (SD), y	34.61 ± 14.05	27.86 ± 11.93	0.001
History of appendectomy, *n* (%)	8 (11.43%)	13 (11.50%)	0.9875
Smoking, *n* (%)	14 (20.00%)	27 (23.89%)	0.5392
Drinking, *n* (%)	10 (14.29%)	13 (11.50%)	0.5812

* t–test or Fisher's exact test is used.

**Table 3:** Clinical characteristics of participants with CD or intestinal BD.

**Characteristics**	**Intestinal BD (*n* = 70)**	**CD** **(*n* = 113)**	*p*–values*
**Clinical manifestations**, ***n*** **(%)**			
Fever	41 (58.57%)	48 (42.48%)	0.0343
Nausea	14 (20.00%)	26 (23.01%)	0.6322
Abdominal pain	64 (91.43%)	99 (87.61%)	0.4211
Diarrhea	22 (31.43%)	77 (68.14%)	<0.0001
Hematochezia	18 (25.71%)	34 (30.09%)	0.5621
Perianal lesions	7 (10.00%)	52 (46.02%)	<0.0001
Anorexia	23 (32.98%)	36 (31.86%)	0.8883
Weight loss	51 (72.86%)	80 (70.80%)	0.7639
**Onset symptoms**, ***n*** **(%)**			
Abdominal pain	57 (81.43%)	66 (58.41%)	0.0013
Diarrhea	16 (22.86%)	39 (34.51%)	0.0946
Perianal lesions	0	20 (17.70%)	0.0002
**Complications**, ***n*** **(%)**			
Abdominal mass	13 (18.57%)	12 (10.62%)	0.1279
Abdominal abscess	3 (4.29%)	7 (6.19%)	0.5808
Intestinal fistula	7 (10.00%)	21 (18.58%)	0.117
Intestinal stenosis	12 (17.14%)	50 (44.25%)	0.0002
Intestinal obstruction	4 (5.71%)	21 (18.58%)	0.0138
Intestinal perforation	7 (10.00%)	2 (1.77%)	0.0123
Intestinal hemorrhage	9 (12.86%)	10 (8.85%)	0.3877
**Extraintestinal Manifestations**, ***n*** **(%)**			
Oral ulcerations	67 (95.71%)	33 (29.20%)	<0.0001
Genital ulceration	28 (40.00%)	2 (1.77%)	<0.0001
Skin lesions	17 (24.29%)	4 (3.54%)	<0.0001
Arthritis	11 (15.71%)	20 (17.70%)	0.7279
Ocular lesions	5 (7.14%)	3 (2.65%)	0.149
Fatty liver	2 (2.86%)	4 (3.54%)	0.801
Cholelithiasis	1 (1.43%)	8 (7.08%)	0.1563
Thromboembolic diseases	5 (7.14%)	1 (0.88%)	0.0312
Myelodysplastic syndrome	6 (8.57%)	0	0.0027

* χ2 test or Fisher's exact test is used.

#### Endoscopic data

Endoscopic features included the lesion site, segmental lesions, ulceration morphology (shallow ulceration, deep ulceration, longitudinal ulceration, irregular ulceration, annular ulceration, oval ulceration, and aphtha), the number of ulcerations (1, 2–5, and >5), ulceration diameter (<5, 5–20, and >20 mm), ileocecal valve involvement, inflammatory polyps, mucosal bridge, and cobblestone appearance (Supplementary Figure 1).

Segmental lesions meant more than two areas with discontinuous ulcerative lesions. Longitudinal ulceration was one with a length longer than 4 cm. If the ulcer occupied more than half of intestinal circumference, we called it an annular ulcer. Aphtha was defined as a tiny, punched out, raised, or flat red lesion with a white center. Cobblestone appearance was defined as a mucosal pattern with raised nodules, resembling the paving of the “Roman” road (4, 10).

All the endoscopic data were read independently by two experienced endoscopists blinded to the patients clinical data, and the final diagnosis was made after discussion if they disagreed with each other.

#### Imaging data collection

Imaging items of CTE included the length of the longest lesion segment, the thickness of the intestinal wall at the site of the lesion, enhancement degree, stratified mural hyperenhancement, asymmetric mural hyperenhancement, polypoid lesion of the mucosal surface, fibrofatty proliferation, and engorged vasa recta (Supplementary Figure 2).

The degree of lesion enhancement was defined according to CT value, with mild enhancement defined as lower than 20 Hounsfield units (HU), moderate enhancement defined at 20–40 HU, and severe enhancement defined as above 40 HU. Stratified mural hyperenhancement was defined as hyperenhancement of the inner (bilaminar) or both the inner and outer (trilaminar) aspects of the bowel wall. Polypoid lesions of the mucosal surface meant protruding and polypoid lesions in the intraluminal surface of the bowel wall. Fibrofatty proliferation, which is also called creeping fat, referred to hypertrophy of the mesenteric fat adjacent to diseased bowel segments with an influx of inflammatory cells and fluid. Engorged vasa recta, which are also known as comb sign, were defined as enlarged blood vessels that supply and drain an inflamed bowel loop (11–14).

All the CTE data were reviewed independently by two experienced radiologists blinded to the patient's clinical data, and the final diagnosis was made after discussion if they disagreed with each other.

### Statistical methods

Categorical variables were described as ratios; the chi-squared test or Fisher's exact test was used for univariate analysis. Continuous variables having a standard normal distribution were presented as mean ± SD; otherwise, they were described as median and interquartile ranges. The *t*-test or the Wilcoxon rank sum test was used for univariate analysis. Variables with a *p*-*value* **<0.0**5 in the univariate analysis were included in a logistic regression model. The receiver operating characteristic (ROC) curve analysis was used to determine the threshold value for continuous variables presenting a linear assumption; otherwise, Locally Weighted Scatterplot Smoothing (LOWESS) function was used. A 10-fold cross-validation method was used to verify the model.

At last, the regression coefficients were divided by the smallest coefficient, and each predictor can get an integer score by rounding. Each patient can have a total risk score by adding the scores of all the predictors. All the data were analyzed by SAS version 9.2 (SAS Institute Incorporation, Cary, North Carolina, USA).

**Figure 1:**
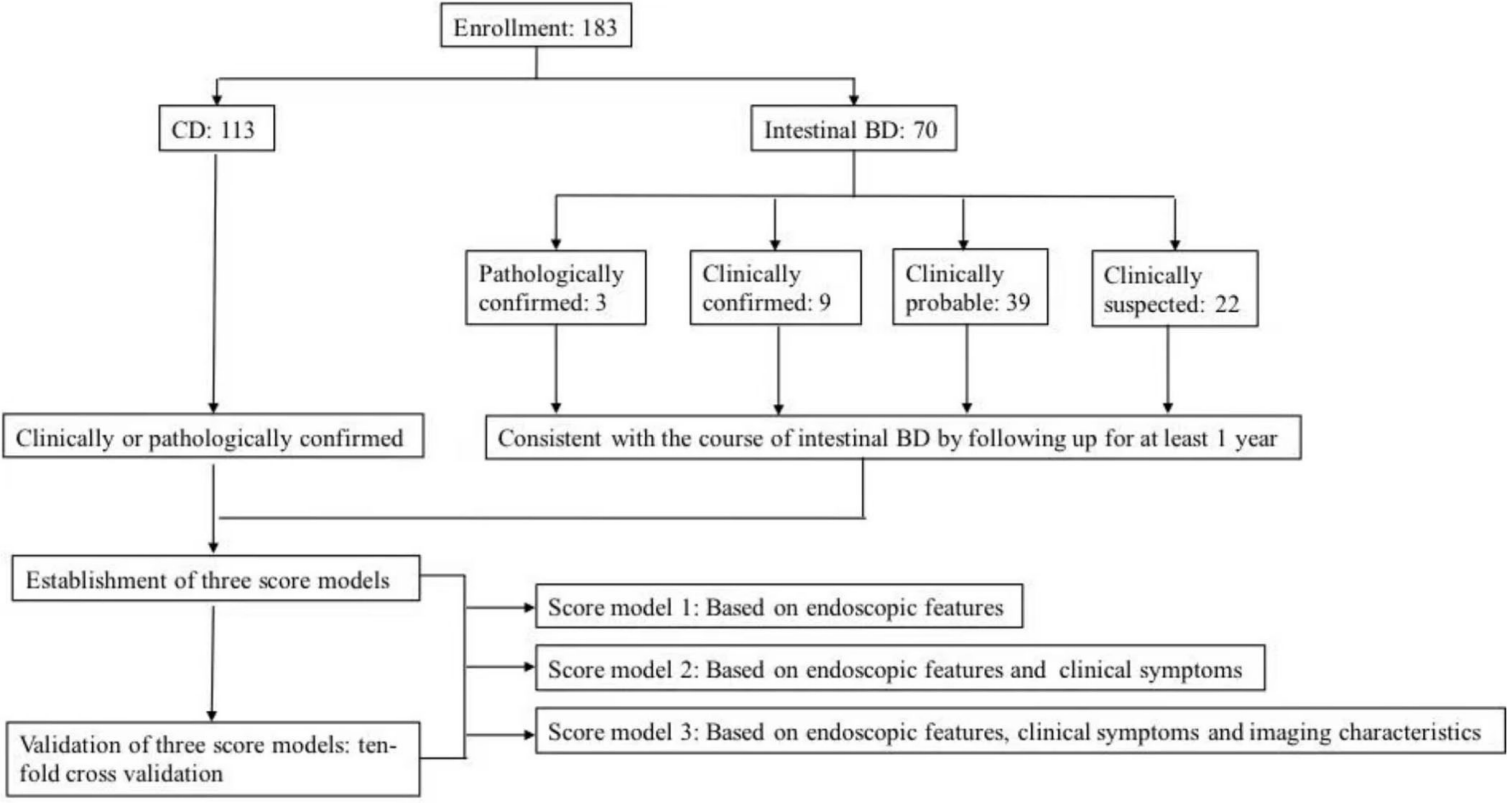
Flow diagram of patient enrollment.

The flow diagram of this study is shown in Figure 1.

## Results

### Demographic characteristics

As seen in Table 2, the percentage of males with CD was higher than that with intestinal BD (*p* < 0.0001). The age of onset of gastrointestinal symptoms for patients with intestinal BD was significantly more than that for patients with CD (*p* = 0.001). However, there were no statistical significance in the history of appendectomy, smoking, or drinking alcohol (*p* **>** 0.05).

### Univariate analysis for potential predictive markers of Crohn's disease and intestinal Behçet's disease

#### Comparative analysis of clinical characteristics

Table 3 shows the differences in clinical features between patients with intestinal BD and patients with CD. In terms of clinical manifestations, patients with intestinal BD were more prone to fever than patients with CD (*p* = 0.0343). The incidence rates of diarrhea and perianal lesions in CD were higher than those in intestinal BD (*p* < 0.0001). There is a slight difference in onset symptoms between the two groups.

In respect of complications, patients with CD were more prone to intestinal stenosis and intestinal obstructions than patients with intestinal BD (*p* **<** 0.05), while patients with intestinal BD were more prone to intestinal perforations (*p* = 0.0123).

In terms of extraintestinal manifestations, patients with intestinal BD had a significantly higher proportion of oral and genital ulcerations than patients with CD (*p* < 0.0001). In addition, skin lesions, thromboembolic diseases, and myelodysplastic syndrome were more common in intestinal BD (*p* < 0.05).

#### Comparative analysis of endoscopic characteristics

Based on colonoscopy, there were statistical significances in the ulceration distribution, type, number, and diameter between the two groups (Table 4). The ileocecal lesion was more common in intestinal BD (*p* < 0.0001). The incidence rates of segmental lesions, longitudinal ulcers, irregular ulcers, and aphtha were higher in patients with CD, while deep ulcers, annular ulcers, and oval ulcers were more common in patients with intestinal BD (*p* < 0.0001). In terms of the number of ulcers, 52.86% of patients with intestinal BD had a single ulcer, and 49.56% of patients with CD had more than five ulcers, showing a significant difference between intestinal BD and CD (*p* < 0.0001). In addition, the incidence rates of inflammatory polyps, mucosal bridge, and cobblestone appearance in patients with CD were higher than those in patients with intestinal BD (*p* < 0.05).

**Table 4:** Endoscopic characteristics of participants with CD or intestinal BD.

**Variables**	**Intestinal BD (*n* = 70)**	**CD (*n* = 113)**	*p*–values*
**Lesion site**			
Terminal ileum	31 (44.29%)	53 (46.90%)	0.7299
Ileocecal region	59 (84.29%)	62 (54.87%)	<0.0001
Ascending colon	18 (25.71%)	46 (40.71%)	0.0387
Transverse colon	8 (11.43%)	42 (37.17%)	0.0001
Descending colon	4 (5.71%)	40 (35.40%)	<0.0001
Sigmoid colon	7 (10.00%)	49 (43.36%)	<0.0001
Rectum	3 (4.29%)	32 (28.32%)	<0.0001
Segmental lesions	11 (15.71%)	79 (69.91%)	<0.0001
**Ulcer morphology**			
Shallow	11 (15.71%)	31 (27.43%)	0.0669
Deep	61 (87.14%)	63 (55.75%)	<0.0001
Longitudinal	4 (5.71%)	45 (39.82%)	<0.0001
Irregular	22 (31.43%)	69 (61.06%)	<0.0001
Annular	21 (30.00%)	6 (5.31%)	<0.0001
Oval	24 (34.29%)	11 (9.73%)	<0.0001
Aphthous	1 (1.43%)	16 (14.16%)	0.0039
**Number of ulcers**			<0.0001
1	37 (52.86%)	27 (23.89%)	
2–5	27 (38.57%)	30 (26.55%)	
>5	6 (8.57%)	56 (49.56%)	
**Ulceration diameter**			0.0027
<5 mm	3 (4.29%)	14 (12.39%)	
5–20 mm	26 (37.14%)	57 (50.44%)	
>20 mm	41 (58.57%)	42 (37.17%)	
Ileocecal valve opening	19 (27.14%)	17 (15.04%)	0.0454
Inflammatory polyps	5 (7.14%)	44 (38.94%)	<0.0001
Mucosal bridge	1 (1.43%)	10 (8.85%)	0.0401
Cobblestone appearance	0	32 (28.32%)	<0.0001

* χ2 test or Fisher's exact test is used.

#### Comparative analysis of the computed tomography enterography features

The results showed (Table 5) that the lesion in patients with CD was longer and thicker compared to patients with intestinal BD. In terms of the enhancement degree of the lesions, the proportion of severe enhancement in patients with intestinal BD was significantly lower than that of patients with CD (*p* < 0.0001). In addition, patients with CD were more likely to have polypoid lesions of the mucosal surface (*p* = 0.0001). Fibrofatty proliferation and engorged vasa recta were also more common in patients with CD (*p* = 0.01 and *p* < 0.0001, respectively).

**Table 5:** CTE characteristics of participants with CD or intestinal BD.

**Variables**	**Intestinal BD (*n* = 70)**	**CD** **(*n* = 113)**	*p*–values*
**Length of the longest lesion segment**			<0.0001
<5 cm	29 (41.43%)	18 (15.93%)	
5–10 cm	26 (37.14%)	31 (27.43%)	
10–30 cm	10 (14.29%)	39 (34.51%)	
>30 cm	5 (7.14%)	25 (22.12%)	
Lesion thickness <1cm	68 (97.14%)	89 (78.76%)	0.0005
**Enhancement degree**			<0.0001
Mild	32 (45.71%)	22 (19.47%)	
Moderate	32 (45.71%)	66 (58.41%)	
Severe	6 (8.57%)	25 (22.12%)	
Stratified mural hyperenhancement	63 (90.00%)	101 (89.38%)	0.8938
Asymmetric mural hyperenhancement	23 (32.86%)	44 (38.94%)	0.4066
Polypoid lesions of mucosal surface	24 (34.29%)	72 (63.72%)	0.0001
Fibrofatty proliferation	31 (44.29%)	72 (63.72%)	0.01
Engorged vasa recta	18 (25.71%)	81 (71.68%)	<0.0001

* χ2 test or Fisher's exact test is used.

### Multivariate analysis to determine independent markers and establishment of score models

In order to compare the abilities of different markers to differentiate CD from intestinal BD, we established three score models based on the multivariate analysis of endoscopic features alone (model 1), combining endoscopic features with clinical manifestations (model 2), and combining CTE and endoscopic features with clinical manifestations (model 3).

#### Differential model 1 by endoscopic data

Model 1 (Table 6) showed that oval ulcer was an independent predictor for intestinal BD (*p* = 0.0027), whereas longitudinal ulcer, number of ulcers >5, and inflammatory polyps were independent predictors for CD (*p* < 0.05). Scores of model 1 are shown in Table 1 according to the method above. According to Youden's index, 1 point was taken as the diagnostic threshold, that is, a total score ≥1 point indicated a diagnosis of CD; otherwise, diagnosis of intestinal BD was indicated. The accuracy of differentiating CD and intestinal BD was 74.32%.

**Table 6:** The multivariate logistic regression analysis and scores of model 1.

**Variable**	**Regression coefficient**	**OR**	**95%CI**	***p*–values**	**Score**
Oval ulcer	−1.4816	0.227	0.086–0.599	0.0027	−1
Longitudinal ulcer	1.7178	5.572	1.665–18.645	0.0053	1
Number of ulcers > 5	2.213	9.143	3.021–27.676	<0.0001	1
Inflammatory polyps	1.513	4.541	1.489–13.849	0.0078	1

#### Differential model 2 by combining endoscopic features with clinical manifestations

Model 2 (Table 7) showed that genital ulcers, skin lesions, and oval ulcers were independent predictors for intestinal BD, while longitudinal ulcers, number of ulcers >5, and inflammatory polyps were independent predictors for CD. With the same methods above, scores of model 2 are shown in Table 2. According to Youden's index, 1 point was taken as the diagnostic threshold, that is, a total score ≥1 point indicated a diagnosis of CD, and a total score <1 point indicated a diagnosis of intestinal BD. The accuracy of the differential diagnosis was 77.60%.

**Table 7:** The multivariate logistic regression analysis and scores of model 2.

**Variable**	**Regression coefficient**	**OR (95%CI)**	***p* values**	**Score**
**Clinical symptoms**				
Genital ulcer	−3.402	0.033 (0.006–0.198)	0.0002	−2
Skin lesion	−2.962	0.052 (0.008–0.326)	0.0016	−2
**Endoscopic features**				
Oval ulcer	−1.873	0.154 (0.048–0.496)	0.0017	−1
Longitudinal ulcer	1.446	4.246 (1.071–16.833)	0.0396	1
Number of ulcers > 5	2.508	12.278 (3.079–48.96)	0.0004	2
Inflammatory polyps	1.782	5.939 (1.471–23.975)	0.0123	1

#### Differential model 3 by infusing computed tomography enterography features into model 2

Model 3 (Table 8) showed that genital ulcers, skin lesions, and oval ulcers were independent predictors for intestinal BD, while longitudinal ulcers, number of ulcers > 5, inflammatory polyps, mucosal hyperenhancement, and fibrofatty proliferation were independent predictors for CD. The scores of model 3 are shown in Table 3. A total score ≥ 2 points indicated a diagnosis of CD; otherwise, a diagnosis of intestinal BD was indicated. The accuracy rate of differentiation was 84.15%.

**Table 8:** The multivariate logistic regression analysis and scores of model 3.

**Variable**	**Regression coefficient**	**OR (95%CI)**	***p* values**	**Score**
**Clinical symptoms**				
Genital ulcer	−3.165	0.042 (0.007–0.238)	0.0003	−3
Skin lesion	−3.389	0.034 (0.004–0.288)	0.002	−3
**Endoscopic features**				
Oval ulcer	−1.826	0.161 (0.045–0.577)	0.005	−2
Longitudinal ulcer	1.375	3.956 (0.950–16.479)	0.0588	1
Number of ulcers > 5	3.070	21.551 (4.512–102.935)	0.0001	3
Inflammatory polyps	1.987	7.292 (1.500–35.462)	0.0138	2
**CTE features**				
Mucosal severe–enhancement	1.937	6.941 (1.638–29.412)	0.0085	2
Fibrofatty proliferation	1.196	3.308 (1.264–8.660)	0.0148	1

**Figure 2:**
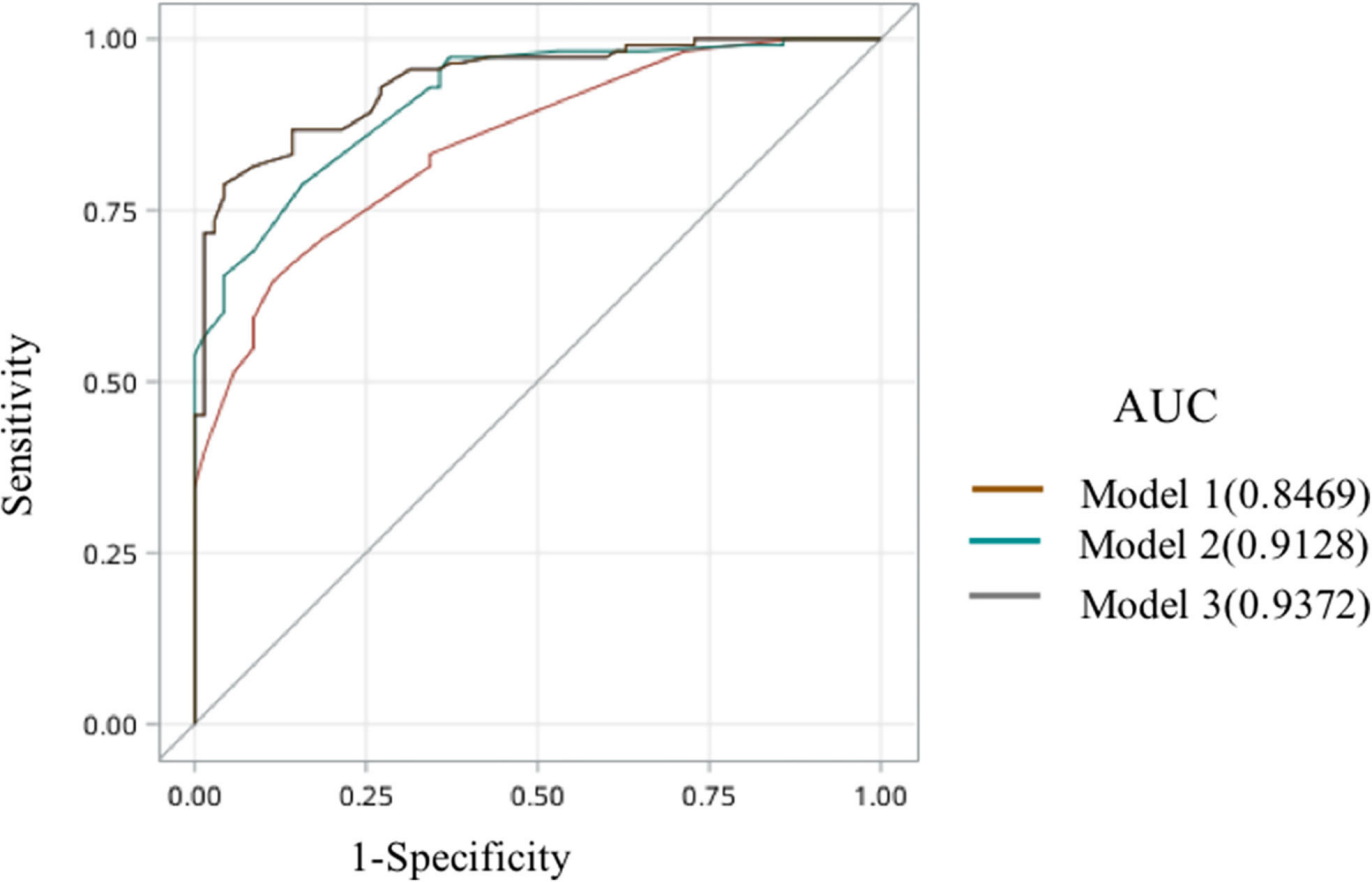
The receiver operating characteristic curve of model 1, model 2, and model 3.

The ROC analysis showed that the area under the curve of model 1, model 2, and model 3 was 0.8469, 0.9128, and 0.9372, respectively (Figure 2). Comparisons among these three models showed that the area under the ROC curve of model 2 was superior to model 1 and that of model 3 was superior to model 2 (*p* **<** 0.05) (Table 9). The goodness of fit of the model could be assessed by the degree of closeness of the calibration curve to the diagonal line and if most of the predicted responses agree with the observed responses, then the blue curve should be close to the diagonal line. In our study, we found that the calibration curve of our model is not too far away from the diagonal line, which implies an acceptable performance of our model in terms of the goodness of fit of the predicted probability, and the calibration plot of model 3 demonstrated good performance (Supplementary Figure 3).

**Table 9:** Comparisons among model 1, model 2, and model 3.

**Models**	**ROC area(AUC)**	**95% CI**	**Accuracy**
Model 1	0.8469	0.7937, 0.9001	0.7432
Model 2	0.9128	0.8740, 0.9517	0.7760
Model 3	0.9372	0.9046, 0.9699	0.8415
Multiple Comparisons,	P		
Model 1 vs. Model 2	0.0021	—	—
Model 2 vs. Model 3	0.0367	—	—
Model 3 vs. Model 1	<0.0001	—	—

### Validation of the score models

The scoring model was verified by 10-fold cross-validation. For these three models, the areas under the ROC curves verified by a 10-fold cross-validation were 0.8072 in model 1, 0.8912 in model 2, and 0.9148 in model 3, as shown in Supplementary Figure 4.

The results indicated that the model based on the clinical, endoscopic, and CTE features had a better ability to distinguish CD from intestinal BD compared to the model based on endoscopic features and clinical symptoms, followed by the model based on endoscopic features alone.

## Discussion

Crohn's disease and intestinal BD have many similarities in clinical characteristics and endoscopic features, but the prognosis of intestinal BD was worse than that of CD; therefore, the differential diagnosis is very important. There are only a few comparative studies about differential diagnosis. In 2009, Lee et al. compared the colonoscopic findings between 115 patients with intestinal BD and 135 patients with CD and developed a simple decision tree differential model based on endoscopic features with a specificity of only 60.3% when applied to Chinese patients, which had limited discriminating value. Compared with previous studies (4, 6), our study found for the first time that clinical symptoms increased the diagnostic accuracy of endoscopic features alone for differentiating CD and intestinal BD, and CTE markers could enhance the diagnostic accuracy of endoscopic and clinical features in the differentiation from 77.60 to 84.15%. Furthermore, we established a scoring model based on 8 items, including genital ulcer, skin lesions, oval ulcer, longitudinal ulcer, number of ulcers >5, inflammatory polyps, mucosal severe enhancement, and fibrofatty proliferation.

In our study, we reassessed the value of the endoscopic model to differentiate CD from intestinal BD. We found that features, such as oval ulceration, longitudinal ulceration, and the number of ulcerations > 5, were still important for differential indices consistent with other studies (4, 6). Besides, we found that inflammatory polyps provided a good differential diagnostic value for the model contributing one point weight for CD diagnosis. However, model 1 based on endoscopic features alone has some limitations for clinical practice. As we know, not all patients have typical endoscopic features. In this study, we found that only 34.29% of patients with intestinal BD appeared with oval ulceration, and only 39.82% of patients with CD appeared with longitudinal ulceration. Therefore, clinical symptoms and CTE features should be considered.

Model 2 was improved by adding clinical symptoms into model 1. We found that clinical symptoms significantly increase the ROC area of model 1 from 0.8469 to 0.9372. Genital ulceration and skin lesions were the two differential symptoms included in model 2. We all know that skin and mucosal lesions were the most characteristic presentations of BD. More than 75% of patients with intestinal BD had genital ulcers, which often occur in the scrotum (15, 16).

In order to enhance the efficacy of the scoring model further, we incorporated imaging features with endoscopic features and clinical symptoms. Compared with model 1 and model 2, model 3 was the best model with an accuracy of 84.15%. Moreover, model 3 in our study was superior to the Korean differential model, including endoscopic features and clinical manifestations, with an accuracy of 78.2%, indicating that CTE features played an important role in the diagnosis of CD and intestinal BD and should be considered in clinical practice. Previous studies showed that mucosal surface severe enhancement is highly correlated with histological activity in patients with CD (17). However, in patients with intestinal BD, because the venules are mainly affected in the gastrointestinal tract (18) and because the inflammatory reaction around the ulcer in patients with intestinal BD is mild (19), it is postulated that the degree of mucosal surface severe enhancement in patients with intestinal BD is not as significant as that in patients with CD. Fibrofatty proliferation, also called “creeping fat,” is a specific manifestation of CTE in patients with CD. In recent years, studies found that “creeping fat” plays an important role in the pathogenesis of CD (20). “Creeping fat” refers to the proliferation of mesenteric adipose tissue around the intestinal wall, which is beyond the normal anatomical range. Such proliferating adipose tissue can secrete inflammatory factors such as tumor necrosis factor-α and transforming growth factor-β, which further cause mesenteric edema and exudation. However, this mechanism was not found in intestinal BD.

The strengths of this study are as follows. First, the scoring methods adopted in this study are based on the regression coefficients of multivariate models. The scoring based on weighing different factors is relatively reasonable and reliable. In addition, this method is simple, convenient, and easy for clinical application. Second, 10-fold cross-validation helps to reduce the overfitting phenomenon. Third, this was a multicenter study, which reduced the selection bias. However, our study also has some limitations. First of all, a prospective study with more patients should be performed to validate these scoring models in the future; second, some laboratory tests, which have the potential differentiating ability such as antiendothelial cell antibodies (AECAs), were not included in this study (21–23).

In conclusion, our results suggest that endoscopic indices combined with CTE examinations and clinical symptoms are of great value in the differentiation of CD and intestinal BD. For hospitals without the ability to perform CTE, clinical symptoms can also increase the differential ability of endoscopic features. It is expected that prospective studies will further validate the models and can be applied in clinical practice.

## Data availability statement

The raw data supporting the conclusions of this article will be made available by the authors, without undue reservation.

## Ethics statement

Written informed consent was obtained from the individual(s), and minor(s)' legal guardian/next of kin, for the publication of any potentially identifiable images or data included in this article.

## Author contributions

HY and JQ: study design, data collection, analysis support, and critical revision of the manuscript. HZ: data collection, analysis, and drafting of the manuscript. WL, WH, TG, YL, BT, CW, MC, XG, ZR, ZL, KW, and QC: data collection. WH: data analysis. All authors contributed to the article and approved the submitted version.

## Funding

This study was supported by the National Nature Science Foundation of China (81570505 and 81970495), the Beijing Municipal Natural Science Foundation (7202161), the Health Research and Special Projects Grant of China (201502005), and the CAMS Innovation Fund for Medical Sciences (CIFMS) (2016-I2M-3-001 and 2019-I2M-2-007).

## Conflict of interest

The authors declare that the research was conducted in the absence of any commercial or financial relationships that could be construed as a potential conflict of interest.

## Publisher's note

All claims expressed in this article are solely those of the authors and do not necessarily represent those of their affiliated organizations, or those of the publisher, the editors and the reviewers. Any product that may be evaluated in this article, or claim that may be made by its manufacturer, is not guaranteed or endorsed by the publisher.
