# Supplementary material for: Computed tomography enterography increases the ability of endoscopy to differentiate Crohn's disease from intestinal Behçet's disease
Source: Front Med (Lausanne). 2022 Aug 18;9:900458. doi: 10.3389/fmed.2022.900458 (PMC9433799; doi:10.3389/fmed.2022.900458)
Supplement: Supplementary file 1 [file Data_Sheet_1.pdf]

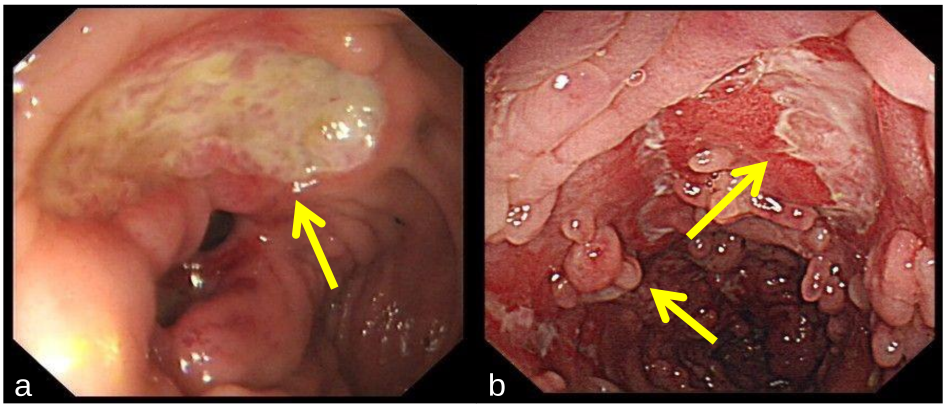

Supplemental Figure 1. Typical endoscopic findings in CD and intestinal BD. **a** The yellow arrow shows an oval ulcer in intestinal BD. **b** The yellow arrows show longitudinal ulcers and inflammatory polyps in CD.

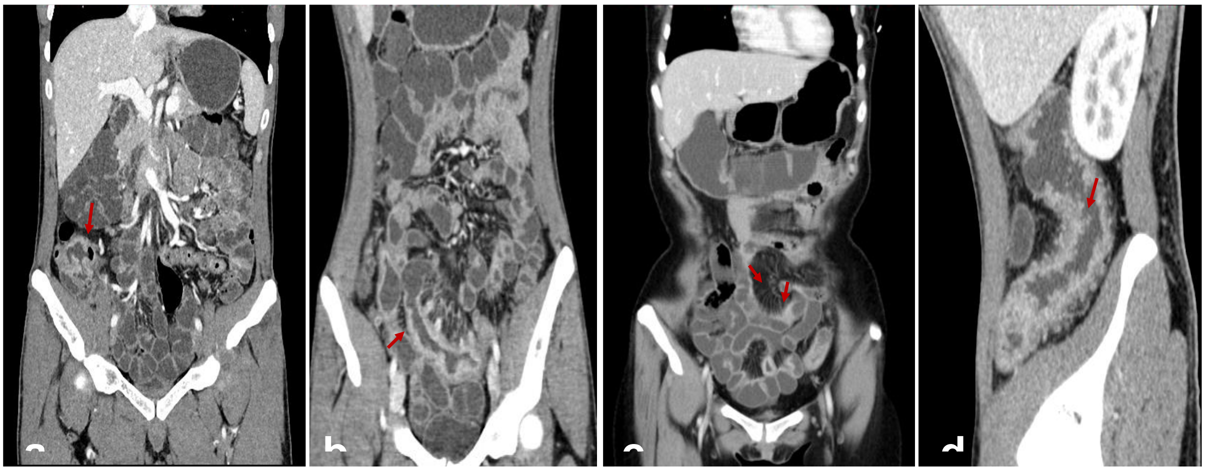

Supplemental Figure 2. Typical CTE findings in CD and intestinal BD. a The red arrow shows mild enhancement of cecum in intestinal BD. b The red arrow shows severe enhancement of ileum in CD. c The red arrows show engorged vasa recta and fatty proliferation in CD. d The red arrow shows polypoid lesion of the mucosal surface in CD.

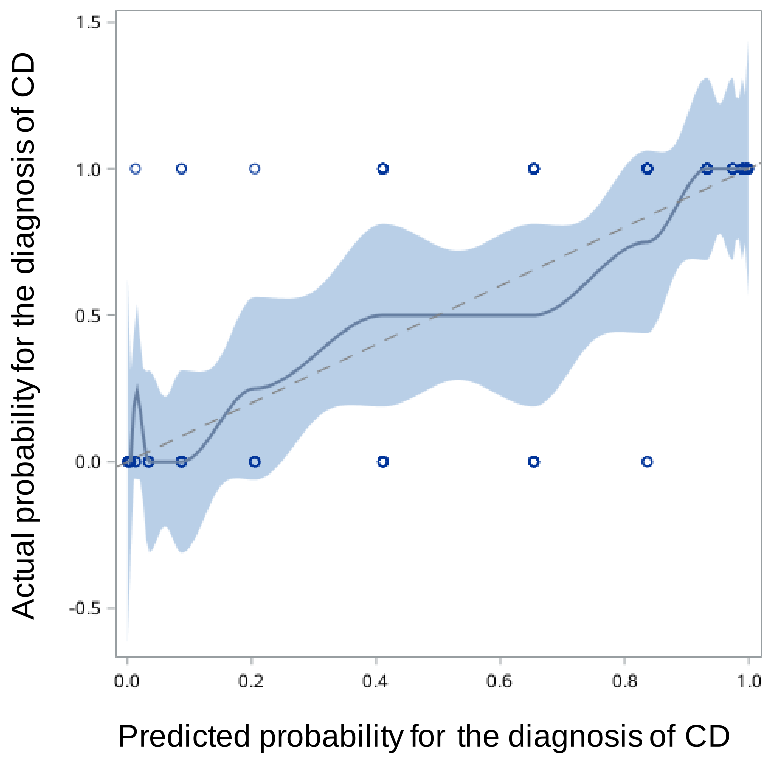

Supplemental Figure 3. Calibration curve for predicting the possibility of CD。The dashed line represents perfect calibration and the blue continuous curve line represents the true frequency of the positive label against its predicted probability, for binned predictions. The light blue band is a 95% confidence region for the loess fit.

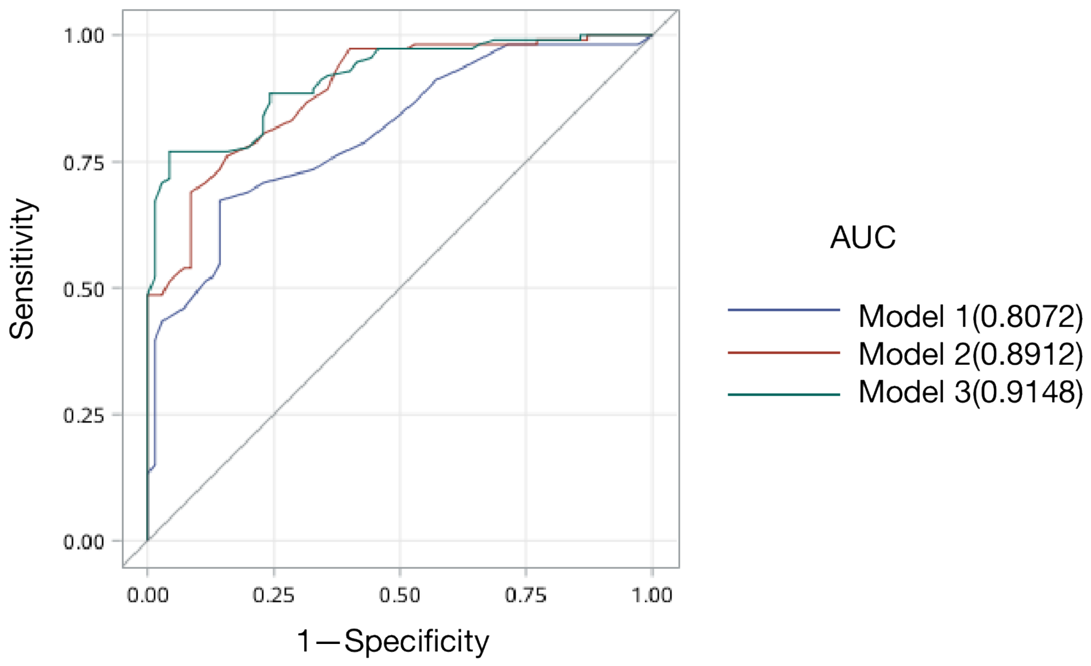

Supplemental Figure 4. Receiver operating curve of model 1, model 2 and model 3 in the validation dataset
